# Supplementary material for: Urine-HILIC: Automated Sample Preparation for Bottom-Up Urinary Proteome Profiling in Clinical Proteomics
Source: Proteomes. 2023 Sep 28;11(4):29. doi: 10.3390/proteomes11040029 (PMC10594433; doi:10.3390/proteomes11040029)
Supplement: Supplementary file 1 [file proteomes-11-00029-s001.zip › Supplementary Figure S1.pdf]

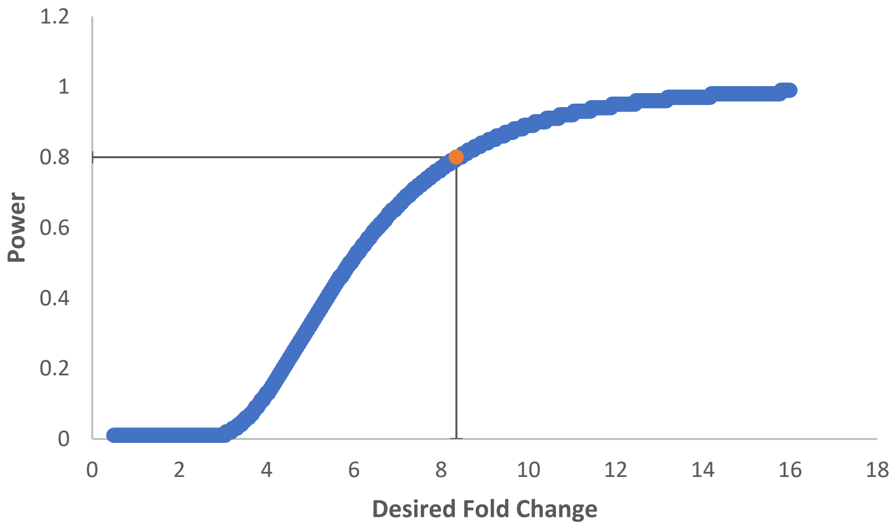

Figure S1: Retrospective power analysis. A fold change of ≥ 8.25 is considered significant at a power of 0.8 with 5 replicates in each group.
